# Combined Cerebellar and Spinal Cord Deficits Caused by an Underlying Gynecologic Malignancy

**DOI:** 10.1155/2020/9021843

**Published:** 2020-01-08

**Authors:** Tamer Othman, Moshe-Samuel Hendizadeh, Ritika Vankina, Susan Park, Phyllis Kim

**Affiliations:** ^1^Department of Internal Medicine, Harbor-UCLA Medical Center, Torrance, CA, USA; ^2^Department of Neurology, Harbor-UCLA Medical Center, Torrance, CA, USA; ^3^Division of Hematology and Medical Oncology, Harbor-UCLA Medical Center, Torrance, CA, USA; ^4^Division of Gynecology Oncology, Harbor-UCLA Medical Center, Torrance, CA, USA; ^5^Department of Hematology and Oncology, Kaiser Permanente Los Angeles Medical Center, Los Angeles, CA, USA

## Abstract

Paraneoplastic cerebellar degeneration (PCD) is an uncommon autoimmune disorder targeting antigens within the nervous system and is usually associated with an underlying malignancy. Neurologic symptoms frequently precede the cancer diagnosis, which is most often seen in women with breast or gynecologic tumors. Anti-Yo-related PCD is the most common PCD syndrome, and one of the best understood. Although cerebellar signs are characteristic of anti-Yo PCD, myelopathy is an unusual presentation of anti-Yo PCD based on published case series and reports. Unfortunately, the prognosis for anti-Yo PCD is often poor, and most patients become bedridden. We report a case highlighting a severe presentation of cerebellar degeneration along with an unusual finding of myelopathy in a patient with a newly diagnosed gynecologic cancer.

## 1. Introduction

PCD results from autoantibodies produced by tumors that target specific antigens within the nervous system [1]. In the case of anti-Yo PCD, the most common form of PCD, the target of the autoantibodies is Purkinje cell cytoplasmic antibody type 1 (PCA1). Anti-Yo PCD accounts for approximately 50% of patients with PCD [2] and is most commonly associated with breast and gynecologic cancers. Thus, females are the majority of patients with this disorder. However, there have been a few cases reported with lung cancers, gastrointestinal, and prostate adenocarcinomas [3–5]. Most commonly, patients present with neurological symptoms, and subsequent laboratory testing and diagnostic imaging reveals an underlying malignancy. Given the rarity of PCD, most of the knowledge of PCD is based off of case series and reports. In this case report, we highlight an unusual presentation of PCD with typical cerebellar signs, as well as myelopathy, in a patient that was found to have positive anti-Yo titers and a gynecologic tumor, which is not typical of this disorder according to the limited literature on the topic. Unfortunately, the patient showed very mild improvement after receiving treatment and physical therapy.

## 2. Case Presentation

A 48-year-old female with no known past medical history presented with a 2-week history of an inability to ambulate, frequent falls, and slurred speech. She denied headaches, diplopia, vertigo, unilateral weakness, sensory changes, bowel or bladder dysfunction, or dysphagia. The neurological exam showed dysarthria, a wide-based ataxic gait, proximal lower extremity weakness, and hyperreflexia in the upper and lower extremities bilaterally with positive cross adductors and Babinski sign, but no Hoffman's sign. Upper and lower limb dysmetria was observed bilaterally. There was no weakness noted in the upper extremities, and normal tone and bulk was seen throughout all extremities. No clonus was observed. Soft touch and pinprick sensation was intact.

Initial basic laboratory studies, MRI of the head and spinal cord, were unrevealing, and no cerebellar atrophy or evidence of myelopathy was seen. Nerve conduction and electromyography (EMG) studies were unremarkable. Serologic workup revealed a positive anti-Yo titer (Table 1). Other assays performed were antiganglioside and antiphospholipid, both of which were negative. In light of the serologic findings, examination of the CSF was deferred. In order to assess for an associated malignancy, a CT abdomen and pelvis was obtained and demonstrated an abnormally enlarged left ovary and enlarged right iliac lymph nodes. The initial CA-125 obtained was elevated to 826.6. A pelvic MRI noted a 4 cm, irregularly shaped and heterogeneously enhancing left ovary, with abnormal marrow changes of the right sacrum and bilateral acetabula, concerning for metastatic disease.

She underwent a bilateral salpingo-oophorectomy with removal of a 4.5 cm pelvic mass near the left fallopian tube. This is typically not the standard of care for ovarian cancer, but it was unclear at the time of surgery if the tumor was gynecologic in origin as it appeared to arise from the mesentery and was connected to the fimbriae. Pathology revealed a serous carcinoma within the fallopian tube and mass. A repeat preoperative CA-125 was elevated to 2,198.4 and decreased to 281.9 post-operation. CT and SPECT after surgery were negative for metastatic disease. She was also found to have a germline BRCA mutation at this time. A diagnostic mammogram demonstrated benign findings.

She was diagnosed with primary peritoneal carcinoma complicated by anti-Yo-related PCD. The Gynecology-Oncology service believed that she likely would not tolerate a complete staging surgery due to her poor performance status and medical comorbidities; thus, the decision was made to proceed with adjuvant chemotherapy since there was no known residual disease. She received one cycle of carboplatin alone due to her poor performance status and postoperative gastroparesis necessitating a gastrojejunostomy tube, followed by 5 cycles of carboplatin and paclitaxel for 6 total cycles of adjuvant chemotherapy with remission confirmed by a nadir in her CA-125. After one and a half years of follow-up, she remains without evidence of disease and is able to move her extremities and transfer with assistance. Other notable changes include a resolution in her gastroparesis and improvement in her speech.

## 3. Discussion

Approximately 90 to 98% of patients who present with cerebellar ataxia and found to have positive anti-Yo antibody titers have an underlying malignancy, often a gynecologic or breast cancer [1]. Anti-Yo-associated PCD typically presents with the subacute development of symptoms characterized by pancerebellar damage, such as severe truncal and limb ataxia [6, 7]. Other symptoms include dysarthria, nystagmus, diplopia, dysphagia, memory loss, emotional lability, and peripheral neuropathy [1, 6]. Myelopathy is primarily a clinical diagnosis diagnosed by clinical exam findings that may include upper and lower motor neuron signs, such as hyperactive reflexes and extremity weakness, and is not regularly described as a finding of anti-Yo-associated PCD [1, 8]. Our patient presented with a combined cerebellar and myelopathic picture, which is normally seen in anti-Hu, anti-Ri, anti-amphiphysin, and GAD65 [8]. EMG was negative for peripheral neuropathy, and MRI of the brain was unrevealing, suggesting that her deficits were due to spinal cord pathology [9].

The exact mechanism of cell death in anti-Yo PCD is not well understood. One proposed mechanism suggests that the coactivation of the humoral immune system and a cytotoxic T-cell response leads to selective death of Purkinje cells. Pathologically, lymphocytic perivascular cuffing, microglial activation, and CD8 lymphocyte infiltration of the cerebellar Purkinje layer are the early changes in PCD, with later changes showing loss of Purkinje cells without inflammation.

There are currently no evidence-based guidelines available for treating anti-Yo-associated PCD. One study showed antitumor therapy to be the only effective method of improving neurological outcomes in these patients [10]. These benefits have not been consistently observed however, and these patients generally have a poor prognosis and are left bedridden with irreversible neurological symptoms. Our case demonstrates that treatment has minimal benefit in relieving neurological symptoms, but some deficits may be recoverable.

## Figures and Tables

**Table 1:** Neoplastic antibody workup.

Anti-Yo (titer)	>1 : 640 (reference range: <1 : 40)
Anti-Ri/Hu	Negative (reference range: negative)
Amphiphysin (titer)	Low levels of antibody detected (reference range: <1 : 100)
NMDAr	Negative (reference range: negative)
Gq1b (titer)	<1 : 100 (reference range: <1 : 100)
MAG (titer)	<1 : 1600 (reference range: <1 : 1600)
GAD65 (IU/mL)	<5 (reference range: <5 IU/mL)
Gliadin (units)	3 (reference range: <20 units)
HTLV1/2	Nonreactive (reference range: nonreactive)
